# Tissue-specific regulatory mechanism of LncRNAs and methylation in sheep adipose and muscle induced by *Allium mongolicum* Regel extracts

**DOI:** 10.1038/s41598-021-88444-9

**Published:** 2021-04-28

**Authors:** Jiangdong Xue, Qi Lv, Erdene Khas, Chen Bai, Bingjie Ma, Wangjiao Li, Qina Cao, Zejun Fan, Changjin Ao

**Affiliations:** 1 Inner Mongolia Key Laboratory of Animal Nutrition and Feed Science, College of Animal Science, Inner Mongolia Agricultural University, Hohhot, 010018 China; 2 College of Animal Science and Technology, Inner Mongolia University for Nationalities, Tongliao, 028000 China; 3 Key Laboratory of Animal Genetics, Breeding and Reproduction in Inner Mongolia Autonomous Region, College of Animal Science, Inner Mongolia Agricultural University, Hohhot, 010018 China

**Keywords:** Non-coding RNAs, Nutrition, Animal physiology, Gene regulation, DNA sequencing, RNA sequencing

## Abstract

*Allium mongolicum* Regel (*A. mongolicum*) is a perennial and xerophytic Liliaceous allium plant in high altitude desert steppe and desert areas. Feeding *A. mongolicum* greatly reduced unpleasant mutton flavor and improves meat quality of sheep. We analyzed epigenetic regulatory mechanisms of water extracts of *A. mongolicum* (WEA) on sheep muscle and adipose using RNA-Seq and whole-genome Bisulfite sequencing. Feeding WEA reduced differentially expressed genes and long non-coding RNAs (lncRNAs) between two tissues but increased differentially methylation regions (DMRs). LncRNA and DMR targets were both involved in ATP binding, ubiquitin, protein kinase binding, regulation of cell proliferation, and related signaling pathways, but not unsaturated fatty acids metabolism. Besides, tissue specific targets were involved in distinct functional annotations, e.g., Golgi membrane and endoplasmic reticulum for muscle lncRNA, oxidative phosphorylation metabolism for adipose lncRNA, dsRNA binding for muscle DMRs. Epigenetic regulatory networks were also discovered to discovered essential co-regulated modules, e.g., co-regulated insulin secretion module (*PDPK1*, *ATP1A2*, *CACNA1S* and *CAMK2D*) in adipose. The results indicated that WEA induced distinct epigenetic regulation on muscle and adipose to diminish transcriptome differences between tissues, which highlights biological functions of *A. mongolicum*, tissue similarity and specificity, as well as regulatory mechanism of mutton odor.

## Introduction

Sheep (Ovis aries) is a typical domestic animal, with a farming history more than 8000 years^1,2^. Along with the movements of nomadic societies, sheep have spread almost globally by a “transportation hub” in eastern Eurasia, Mongolian Plateau region^3,4^. This type of ruminants has played an important role in human society, and is a major source of meat, milk, and fiber in the form of wool^5–7^. As a unique and necessary food source for the human diet, mutton has abundant nutrients, such as iron, zinc, selenium, fatty acids and vitamins^8^. Mutton consumption in north China (e.g., in Inner Mongolia), is more popular than that in other regions, because meat flavor and odor is more pleasant than that in other regions, especially for older sheep^9^. Mutton odor originates from two types of branched chain fatty acids (BCFAs): 4-methyl nonanoic acid (MNA) and 4-ethyloctanoic acid (EOA) present in all the adipose tissue; and the 3-methylindole (MI), skatole or indole originated from pastoral diets. Researchers have been focusing on meat quality of sheep adipose and muscle^10^. For instance, storage of uncured mutton adipose tissue samples under anoxic conditions limits lipid oxidation but enhances mutton odor intensity^11^. Sheep natural mutations that affect muscle growth (e.g., callipyge, *CLPG*) have potential to significantly improve lamb-meat quality^12^. In sheep muscle, some motifs are mutated and bind to corresponding transcriptional factors to regulate promoter activity and musclin gene expression^13^. Dietary improves nutritional value and meat quality of mutton, e.g., dietary vitamin E increases polyunsaturated fatty acid (PUFA) content and decreases saturated fatty acid (SFA) content of mutton^14^.

*Allium mongolicum* Regel (*A. mongolicum*, also known as Mongolia leek) is a type of perennial and xerophytic *Liliaceous allium* plant in high altitude desert steppe and desert areas and is considered as one specific food source for pleasant meat flavor^15,16^. This plant is involved in multiple biological and physiological processes. For instance, polysaccharides extracted from *A. mongolicum* affect signaling molecules of sheep peripheral blood lymphocytes in vitro by regulating the concentrations of Ca^2+^, NO, cAMP and cGMP^17^. *A. mongolicum* and its extracts increase the average daily gain (ADG), feed intake and feed remuneration of broiler chickens through growth-related hormones^18,19^. However, the underlying regulatory mechanism of how this plant effectively eliminates mutton odor in sheep is still unknown.

Epigenetics are heritable molecular determinants of phenotype by adjusting gene expression without having changes in DNA sequences. Epigenetic mechanisms are implicated during development in utero and at the cellular level, and bioactive food components may trigger protective epigenetic modifications throughout life^20^. Epigenetic features include DNA methylation, long non-coding RNAs (lncRNAs), histone modifications, microRNA expression, and chromatin structure, which regulate gene expression through distinct mechanisms^21,22^. For instance, DNA methylation represses gene expressions by interfering the normal function of transcriptional activators on promoters or the repeated sequences across gene bodies^23,24^; while lncRNAs influence expression or stability of protein-coding RNAs in a localized, gene-specific fashion or by targeting large chromosomal regions^25,26^.

One characteristic of these epigenetic markers is tissue specificity, e.g., muscle and adipose tissues. There is evidence that environmental exposures during early life can induce persistent alterations in the epigenome, which may have subsequent effects on disorder of adipose later in life^27,28^. Specifically, DNA methylation plays an important role in several differentiation processes and possibly in adipocyte differentiation, e.g., WGEF expression regulates adipogenesis through DNA methylation change in 3T3-L1 cells^29^. Besides, muscle development is also regulated with epigenetic modification. Genes important for myogenesis are up regulated by histone acetyltransferases and other chromatin modifiers correlated with several transcription factors, including Pax7, MyoD and Mef2^30^. In addition, 1407 differentially expressed lncRNAs that showed consistent expression patterns between skeletal muscles of Large White pigs and Mashen pigs, indicating the involvement of lncRNAs in muscle development^31^. Moreover, phosphotyrosine phosphatase inhibitor bisperoxovanadium resets the cell fate program by specific epigenetic regulations, which leads to an enhanced regenerative potential of muscle stem cell^32^. Therefore, understanding epigenetic mechanism of feeds is essential for muscle and adipose in animal production.

In this study, we applied whole-genome Bisulfite sequencing and RNA-sequencing to analyze epigenetic changes in adipose and muscle induced by water extracts of *A. mongolicum* (WEA). We systematically investigated alterations of transcriptome and epigenome and studied potential epigenetic mechanism by target genes regulated by lncRNAs and methylation, which highlights significance of *A. mongolicum* as a unique sheep feed to improve meat quality.

## Results

### High quality of transcriptome sequencing data

**Figure 1:**
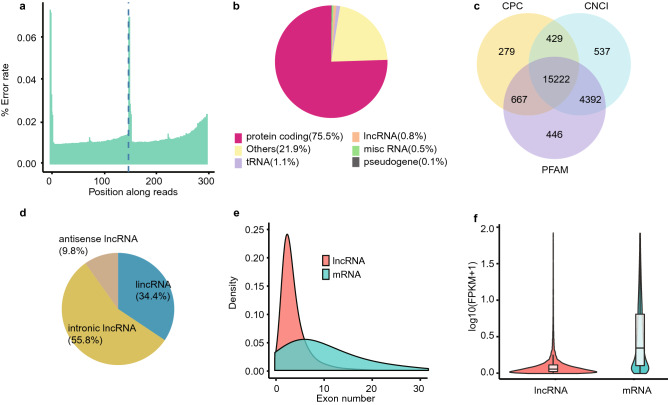
Analyses of mRNA and lncRNA from RNA-seq data. (**a**) Sequencing error rates of read base positions from 1 to 300 bp. (**b**) Percentages of protein coding RNA, lncRNA, misc RNA, rTNA, pseudogene and other classifications for all the mapped reads. (**c**) Comparisons of candidate lncRNAs identified by CPC, CNCI and PFAM. (**d**) Proportions of three lncRNA classifications of lincRNA, antisense lncRNA and intronic lncRNA. (**e**) Density distributions of exon number for both mRNA and lncRNA. (**f**) FPKM distribution of both lncRNA and mRNA.

We obtained 15.08G ± 1.23G clean reads after quality control, and quality scores of over 93.24% reads were more than Q30. Maximum error rate was less than 0.08%, indicating high quality of sequencing data (Table S1, Fig. 1a). Over 81.05% clean reads were mapped to genome, including over 68.45% uniquely mapping (Table S2). Reads were mainly mapped to protein coding regions (73.55% ± 2.88%) and lncRNAs (0.72% ± 0.12%) (Fig. 1b). 324,356 transcripts were assembled by StringTie, including 43,674 protein coding transcripts. Remaining transcripts were analyzed with exon number, transcript length, known annotation, expression level and protein coding potential (see methods). Finally, 15,222 transcripts with non-coding potential were predicted by CPC, CNCI and PfamScan, and defined as the lncRNAs (Fig. 1c). These lncRNAs were classified into intronic lncRNAs (55.8%), intergenic lncRNAs (34.4%) and anti-sense lncRNAs (9.8%) (Fig. 1d). Compared with mRNA, lncRNAs had less exon numbers, shorter sequence lengths and open reading frame (ORF) lengths (Fig. 1e, Figure S1a, S1b). Distributions of overall mRNA expressions were consistent among 12 samples (Figure S1c). In addition, the expressions of different treatments of the same tissues were of high correlation coefficient (0.877 ± 0.011), higher than that between different tissues (0.670 ± 0.022) (Figure S2). Expression of lncRNAs was lower than mRNA expression (Fig. 1f).

### Methylation characteristics distinguished by methylation context

**Figure 2:**
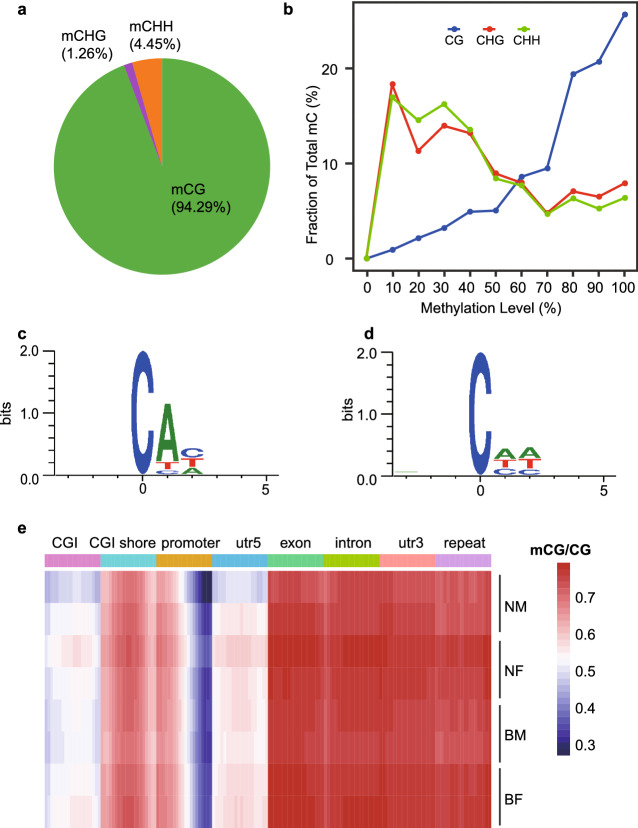
Methylation characteristics of CG, CHG and CHH contexts. (**a**) Proportions of methylated C sites in CG, CHG and CHH contexts. (**b**) Fractions of methylation levels in CG, CHG and CHH contexts, respectively. (c) Methylation motif in CHG context. (d) Methylation motif in CHH context. (e) Methylation ratios of the whole CG context in CGI, CGI shore, promoter, utr5, exon, intron, utr3 and repeat regions for normal muscle (BM), normal adipose (BF), muscle induced by WEA (NM) and adipose induced by WEA (NF) samples.

Average Bisulfite sequencing depth of bases was 17.70 ± 0.49, while sequencing coverages of bases (sequencing depth > 10 ×) were 66.42–78.82% (Table S3). Totally we obtained 42.56 Gb C sites and average coverage depth was 5.03 ± 0.20 (Table S4). Base numbers were 267.98 ± 12.69 (Mb), 1309.16 ± 45.64 (Mb) and 3870.56 ± 148.42 (Mb) for CG, CHG and CHH respectively (Table S4). Average methylation level of CG was 70.60% ± 1.59%, while that of CHG and CHH were only 0.27% ± 0.02% and 0.27% ± 0.02% (Table S4). Over 30.01% of C sites were methylated in CG context, which was higher than that in the whole genome context, CHG or CHH (> 1.67%, > 0.09%, > 0.16%). 92.17% ± 4.74% of methylated C sites was in CG context, while 6.52% ± 4.85% was in CHH context. For example, in normal adipose tissue, percentages of mCG, mCHG and mCHH were 94.29%, 1.26% and 4.45% (Fig. 2a). Methylation levels in CG context were higher than other context, e.g., in normal adipose tissue (Fig. 2b). Methylation sites in CHH context were mainly CAC (Fig. 2c), which was different from that in the whole genome sites (Fig. 2d). In CG context, single-sample C site methylation level was from 31.82% ± 7.80% to 82.72% ± 1.19%, and single-sample C site methylation density was from 86.12% ± 1.30% to 98.64% ± 0.29%, which were higher than that in CHH context and CHG context (Table S5). Methylation levels in CG context was consistent in eight gene functional regions among samples (Fig. 2e).

### Distinct transcriptome and methylation alterations between two tissues induced by WEA

**Figure 3:**
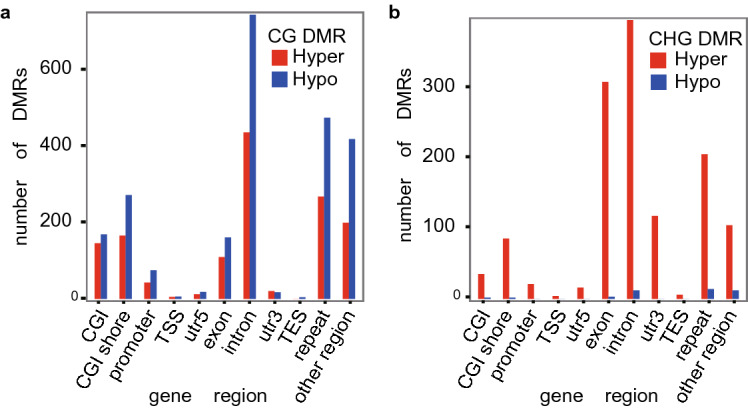
Comparisons of hyper (high) and hypo (low) methylation DMRs against gene regions in CG and CHG methylation contexts in adipose. (**a**) Frequency of hyper CG and hypo CG methylation in CGI, CGI shore, promoter, TSS, utr5, exon, intron, utr3, TES, repeat and other gene regions. (**b**) Frequency of hyper CHG and hypo CHG methylation in CGI, CGI shore, promoter, TSS, utr5, exon, intron, utr3, TES, repeat and other gene regions.

We first examined influence of WEA specific for adipose. WEA induced 4160 differentially expressed mRNAs (2854 up-regulated and 1306 down-regulated) related to 2842 unique DEGs (Figure S3), and 1171 DElncRNAs including 842 up-regulated and 329 down-regulated lncRNAs. WEA increased average CHH and CHG methylation levels at CGI shore region, beginning of promoter region, exon region, intron region and 3′utr region (Figure S4). We identified 19,983 DMRs in adipose induced by WEA. Average DMRs length was 160–180 bp in CHH context, while that was 80–90 bp in CG and CHG context (Figure S5a, S5b, S5c). These DMRs included 17,751 hyper-methylation regions and 2232 hypo-methylation regions. The number of CG hypo-methylation regions was more than the number of CG hyper-methylation regions in intron, repeat and CGI shore (Fig. 3a). However, CHH and CHG DMRs were mainly hyper-methylation, and had no difference among gene regions (Fig. 3b, Figure S6).

**Figure 4:**
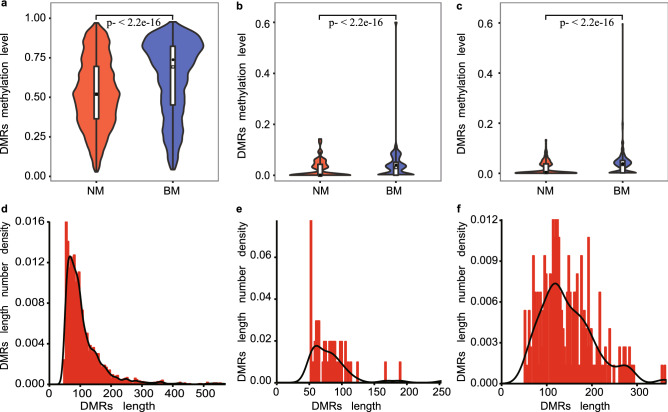
Methylation levels and length number densities of CG, CHG and CHH context DMRs in muscle. (**a**) Violin plot of DMRs methylation level in CG context (*p* value < 2.2e−16). (**b**) Violin plot of DMRs methylation level in CHG context (*p* value < 2.2e−16). (**c**) Violin plot of DMRs methylation level in CHH context (*p* value < 2.2e−16). (**d**) DMRs length number density in CG context. (**e**) DMRs length number density in CHG context. (**f**) DMRs length number density in CHH context.

In muscle, WEA induced 4,694 differentially expressed mRNAs in muscle (Figure S7), including 2703 up-regulated and 1991 down-regulated mRNAs related to 3092 DEGs, 1412 DElncRNAs including 687 up-regulated and 725 down-regulated lncRNAs. Similar with adipose, the overall methylation levels of muscle were higher in gene body and downstream 2 kb region than that in other gene regions (Figure S8a). In addition, WEA decreased methylation levels in CG, CHG and CHH context (Fig. 4a–c). 5019 DMRs were induced by WEA, including 2787 hyper-methylation regions and 2232 hypo-methylation regions. Average lengths of CG DMRs and CHG DMRs were 60–70 bp (Fig. 4d, e). However, average length of CHH DMR was 110–120 bp (Fig. 4f), which was shorter than that in adipose. DMRs in muscle included more hypo-methylation regions in CG, CHG and CHH context (Figure S8b, S8c, S8d).

### WEA decreased transcriptome differences and increased methylation differences between adipose and muscle

WEA reduced transcriptional differences between muscle and adipose. In normal tissues, we discovered 107 differentially expressed mRNAs corresponding to 99 genes. Expression of 95 mRNAs were higher in muscle than in adipose (Figure S9). After WEA treatment, DEGs number was decreased to only 3 (*CACNG6*; *DUSP13*; and *105,606,075* gene, Figure S10), indicating that WEA reduced transcriptome differences between muscle and adipose. In PCA analysis of 12 samples from the four experimental groups (Figure S11), we discovered that in the first principal component (PC1) WEA reduced the PCA space between mutton muscle and adipose tissues, while the tissues differences were still maintained. In addition, the number of DElncRNAs was also decreased from 9 to 1. Effects of WEA on methylation between tissues were different from transcriptome. In normal tissues, average length of tissue CG DMRs was 110 bp (Figure S12a). 9,760 DMRs were discovered, including 2339 hyper- and 7421 hypo-methylation regions. Hypo- and hyper-methylation distributions on gene region were different in CG, CHH and CHG context. The numbers of CG hypo-methylation were more than that of CG hyper-methylation in intro and repeat regions (Figure S12b), while more CHG and CHH hypo-methylation existed in all gene regions (Figure S12c, S12d).

**Figure 5:**
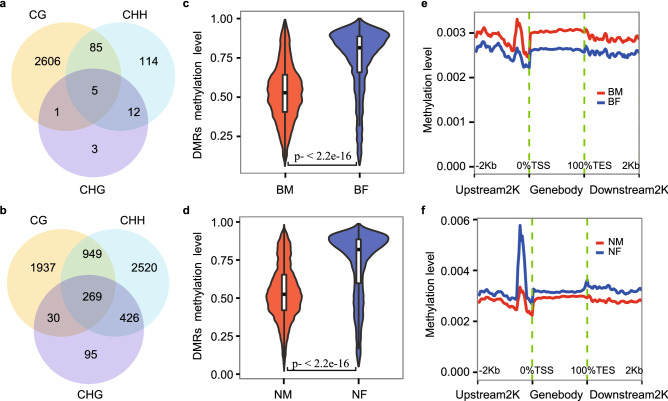
Effects of WEA on methylation difference between adipose and muscle. (**a**) Venn diagram of CG, CHG and CHH DMR genes in muscle. (**b**) Venn diagram of CG, CHG and CHH DMR genes in adipose. (**c**) Comparisons of CG DMRs methylation levels for normal muscle (BM) and adipose (BF) (*p* value < 2.2e−16). (**d**) CG DMRs methylation levels for muscle (NM) and adipose (NF) induced by WEA (*p* value < 2.2e−16). (**e**) Comparison of CHG and CHH methylation levels against upstream 2 K, gene body and downstream 2 K for normal muscle and adipose. (**f**) Comparison of CHG and CHH methylation level against upstream 2 K, gene body and downstream 2 K for muscle and adipose induced by WEA.

Interestingly, compared with 2826 DMR genes between normal tissues, the number of DMR genes between treatment tissues was increased to 6226, indicating that WEA induced more DMR. WEA’s effects on methylation were different to the decreasing tissue differences of transcriptome and lncRNAs. DMR genes showed distinct patterns among three methylation contexts. In normal tissues, we discovered 2,697 CG DMRs genes and 216 CHH DMRs genes, which had 90 genes in common (Fig. 5a). However, WEA increased the numbers of CG and CHH DMRs genes to 3185 and 4164 (Fig. 5b). Overall CG methylation levels in normal conditions showed higher methylation levels in adipose (Fig. 5c) and were not affected by scallions (Fig. 5d). By contrast, the CHH and CHG methylation levels of muscle were higher than that in adipose under normal condition (Fig. 5e, Figure S13a), while WEA feeding induced higher CHH and CHG methylation levels in adipose (Fig. 5f, S13b).

### Functional interpretation of DMRs genes

Then, we did pathway and GO enrichment analyses for DMRs genes to investigate the underlying regulatory mechanism of increasing methylation difference between two tissues. Results indicated that the DMRs genes were mainly involved in energy metabolism, signaling pathways and cell proliferation.

**Figure 6:**
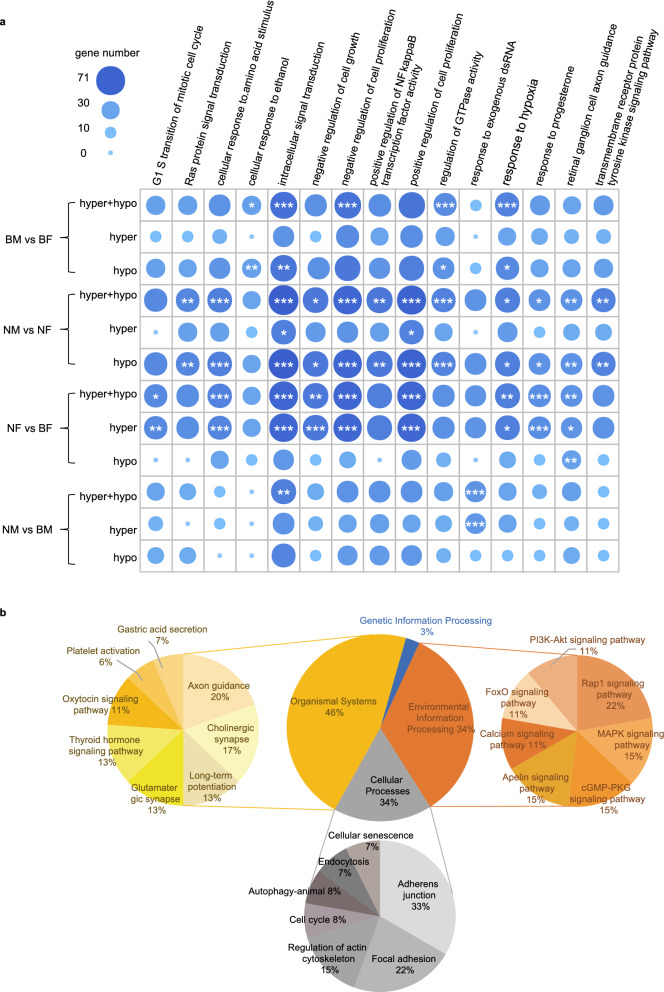
Functional enrichment for DMRs genes. (**a**) 15 enriched biological process GO terms of different methylation levels for normal tissue comparison (BM vs BF), tissue comparison induced by WEA (NM vs NF), effects of WEA on adipose (NF vs BF) and muscle (NM vs BM). *Adjusted *p* value < 0.05, **adjusted *p* value < 0.01, ***adjusted *p* value < 0.001. (**b**) Percentage of enriched KEGG pathways for different methylation levels for normal tissue comparison, tissue comparison induced by WEA, effects of WEA on adipose and muscle.

DMRs genes induced by WEA were significantly enriched in 21 GO terms, while that between normal tissues were only 7 GO terms (adjusted *P* < 0.01, Fig. 6a, Figure S14, Figure S15). 4 GO terms (ATP binding, intracellular signal transduction, negative regulation of cell proliferation and regulation of GTPase activity) were related to tissue difference and not affected by WEA treatment. The remaining 17 GO terms were induced by WEA treatment and were mainly about signaling transduction and signaling related responses, e.g., transmembrane receptor protein tyrosine kinase signaling pathway, Ras protein signal transduction, positive regulation of NF-kappaB transcription factor activity, transmembrane receptor protein tyrosine kinase activity, protein kinase binding, phosphotyrosine residue binding, postsynaptic membrane, focal adhesion, cytoskeleton and caveola. Interestingly, ubiquitin protein ligase binding, nucleoplasm and positive regulation of cell proliferation were also identified as enriched GO annotations here, indicating their essentiality in tissue differences regulated by WEA. Methylation alterations of these GO were all hypo-methylation (Fig. 6a, Figure S14, Figure S15), indicating WEA induced higher methylation level of these GO terms in adipose than that in muscle.

In addition to tissue differences, we discovered tissue specific functional annotation of DMRs genes (see NF VS BF, NM VS BM in Fig. 6a, Figure S14, Figure S15). In adipose, enriched GO terms were also related to the signaling pathways and cell proliferation. In muscle, we found four enriched GO annotations, including intracellular signal transduction, ATP binding, response to exogenous dsRNA and double stranded RNA binding. dsRNA was only enriched in hyper DMR genes, indicating that WEA might regulate dsRNA specifically in muscle through enhancing methylation of related genes. Compared with adipose, enriched GO annotations in muscle were less than that in adipose.

Pathway enrichment indicated that the effects of WEA on hyper-, hypo- and overall methylation were about genetic information processing, environmental information processing, cellular processes and organismal systems, but not about fatty acid metabolic pathways (Fig. 6b). And the results were similar with GO, e.g., ubiquitin mediated proteolysis, Rap1 signaling pathway, MAPK signaling pathway, axon guidance, cholinergic synapse, adherens junction, focal adhesion, regulation of actin cytoskeleton, cell cycle, etc.

### Functional annotation of lncRNAs’ targets induced by WEA

We discovered that WEA induced 30,911 lncRNA-target pairs of 1218 lncRNAs (86.26% of total) and 2296 DEGs (74.25% of total) in muscle, while in adipose there were 24,483 pairs including 974 lncRNAs (83.18% of total) and 2035 DEGs (71.60% of total) (Methods). Results of normal tissue comparison were less, with only 359 lncRNA-target pairs including all the 9 DElncRNAs and 87 DEGs (81.31% of total).

LncRNAs targets were significantly enriched in GO terms related to energy metabolism, signaling transduction and cell proliferation, which were the same with the results of methylation, e.g., NAD binding, npBAF complex, NAD + kinase activity, pyridoxal phosphate binding, etc. Effects on signaling transduction were mainly about magnesium ion binding, protein kinase binding, growth hormone receptor binding, lipopolysaccharide receptor activity, regulation of cytokine secretion, etc. Furthermore, these targets were also significantly enriched in transcription regulation, e.g., ubiquitin protein ligase binding, ubiquitin conjugating enzyme activity, SWI/SNF complex and transcription factor TFIID complex. In addition, lncRNAs target were also enriched in positive regulation of cell proliferation. Besides, we discovered that lncRNAs targets were correlated with macrophage activation (a biological process related to immunity).

lncRNAs targets showed tissue differences in environmental information processing, cellular processes, metabolism and organismal systems pathways. For instance, the targets in muscle were significantly enriched in protein synthesis related GO terms, e.g., endoplasmic reticulum unfolded protein response, Golgi membrane, endoplasmic reticulum. These enriched GO terms were specific identified in muscle, which was distinguished from adipose. A metabolism pathway (oxidative phosphorylation pathway) was notably specific for lncRNA regulation in adipose, which was different from the results of methylation. Besides, other enriched pathways were the same with the results of methylation, e.g., ubiquitin mediated proteolysis, endocytosis, apelin signaling pathway, cGMP-PKG signaling pathway, focal adhesion, etc.

### Essential targets in co-regulated module of epigenetic regulatory networks

**Figure 7:**
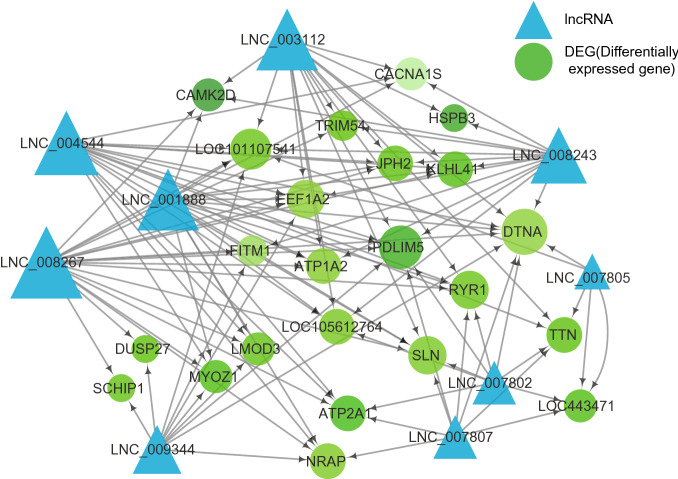
The co-regulatory network of both lncRNA and methylation for normal adipose and muscle.

By integrating both methylation and lncRNA results, we constructed and analyzed the regulatory networks of tissue difference or specific tissue to discover essential regulators and genes during transcriptional processes. We obtained a regulatory network of tissue difference between normal muscle and adipose (Fig. 7), including nine differentially expressed lncRNAs (Table S6) and 23 methylated target genes. Under the regulation of lncRNAs and methylation, the expressions of all the target genes were decreased in muscle. Each lncRNA regulated seven target genes on average.

Besides, we discovered that WEA induced complex networks for specific tissue on account of more regulators and more target genes. The regulatory network for muscle had 983 regulatory interactions including 517 differential lncRNAs and 104 target genes (Figure S16). The expressions of 68 target genes were up-regulated by the regulation of both lncRNAs and methylation, while the remaining 36 genes were down-regulated. Interestingly, we uncovered that co-regulated genes in the regulatory network, that were regulated by the same lncRNAs. For instance, *MAPKAP1* gene and *FOXP2* gene were co-regulated genes. *MAPKAP1* gene was regulated by 135 *lncRNAs*, while *FOXP2* gene were regulated by 121 lncRNAs. 108 of *MAPKAP1* gene regulators (80.00%) were the same with the regulators of *FOXP2* gene (89.26%). In another example, *STXBP5* gene and *WDR17* gene were regulated by 46 and 40 lncRNAs respectively. 35 lncRNAs were the same, accounting for 76.09% and 87.50% individually. These co-regulated gene pairs shared by gene pairs suggested that the intake of WEA induced similar downstream regulation mechanism of lncRNAs, which could be essential regulators.

Similar with the regulatory network of muscle, the regulatory network of adipose also suggested complex regulatory mechanism (Figure S17). This network included 346 lncRNAs and 80 target genes. 59 of the target genes were up-regulated, while the remaining 21 were down-regulated. In adipose, co-regulated modules contained more than two target genes, that was different from the co-regulated modules in muscle. In one co-regulated module, seven target genes (*THSD4*, *SLC9A7*, *PDPK1*, *GMDS*, *DLG4*, *CDH4* and *ADGRG6*) were co-regulated by 18 lncRNAs, accounting for more than 31.58% of lncRNAs in regulatory network. In another example, *SLC25A12*, *MAP2K6*, *REEP1*, *MYBPHL*, *CLCN1* and *PHB* were co-regulated by six lncRNAs. The co-regulation of target genes in these modules suggested that DEGs induced by WEA had high function correlation in adipose.

### Validating key target genes in epigenetic regulatory networks by RT-PCR

**Figure 8:**
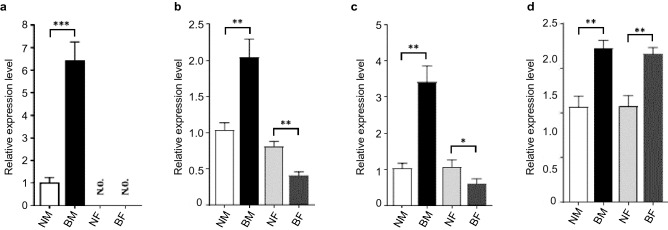
Expressions of *MYOZ1* (**a**), *PDLIM5* (**b**), *LMO7* (**c**), and *TFRC* (**d**), in muscle and adipose samples. (*p value < 0.05, **p value < 0.01, ***p value < 0.001).

We randomly selected some target genes from epigenetic regulatory network and examined their expressions by fluorescence quantitative PCR (Fig. 8) to validate high-throughput results. *MYOZ1* was a key target gene regulated by eight lncRNAs (LNC_001888, LNC_003112, LNC_004544, LNC_007802, LNC_007807, LNC_008243, LNC_008267 and LNC_009344) and methylation. *MYOZ1* expression was specific for muscle, which was reduced by WEA. *PDLIM5* was regulated by methylation and four lncRNAs (LNC_001888, LNC_004544, LNC_008267 and LNC_009344). *PDLIM5* was expressed both in muscle and adipose. However, WEA effects on *PDLIM5* expression were distinct in the two tissues. WEA increased *PDLIM5* expression in adipose, while decreased *PDLIM5* expression in muscle, suggesting different biological functions of *PDLIM5* in the two tissues. Results of *LMO7* were similar with *PDLIM5*, revealing potential functional correlations between *LMO7* and *PDLIM5*. Expression patterns of *TFRC* were different from the above three target genes. *TFRC* expressions were reduced by WEA, which was the same in two tissues, indicating that biological functions of *TFRC* were not specific to muscle or adipose. Expressions of the four target genes in fluorescence quantitative PCR were in accordance with RNA-seq results, suggesting possible distinct biological functions of target genes in epigenetic regulatory networks.

## Discussion

In this study, we analyzed epigenetic effects of WEA on muscle and adipose tissues with both methylation and transcriptome data to uncover how this plant reduce the unpleasant flavor of mutton. The results provided an overview of the whole genome changes induced by WEA and highlighted the underlying regulatory mechanisms of methylation and lncRNAs.

Here, we discovered that WEA reduced DEGs number of normal tissues to only 3 (*CACNG6*, *DUSP13* and *105,606,075*), suggesting that transcription differences of genes were minimized to maintain tissue specificity. These three DEGs induced by WEA might be essential genes in remaining tissue specificity. *CACNG1* and *DUSP13* were reported to be closely correlated with muscle in animals. *CACNG1* (Voltage-dependent calcium channel gamma-1 subunit) encodes a muscle-specific isoform of the Ca^2+^ channel gamma subunit. *CACNG1* is increased in the lamina propria of the old rats, and the deleterious mutation is discovered in a young patient with metastatic urothelial carcinoma^33,34^. *DUSP13* (Dual specificity protein phosphatase 13) encodes two atypical DUSPs, DUSP13B/TMDP and DUSP13A/MDSP, that are specifically expressed in testis and skeletal muscle respectively^35,36^. In addition to the known biological function of *CACNG1* and *DUSP13*, our results provide more evidence for further understanding genes functions to maintain tissue characteristics under the regulation of WEA in sheep.

We discovered that the regulatory changes of lncRNAs and methylation had different patterns: decreased differences in lncRNAs and increasing differences in methylation, indicating that WEA had distinct influences on lncRNAs and methylation regulation. Targets of lncRNAs and methylation were both significantly enriched in energy metabolism, signaling pathways and cell proliferation related annotations, e.g., ATP binding, ubiquitin protein ligase binding, protein kinase binding, positive regulation of cell proliferation, focal adhesion, etc. These annotations were closely correlated with muscle and adipose physiology. For instance, focal adhesion is a dynamic cellular process in cell proliferation and migration, and the phosphorylation of focal adhesion molecules is significantly reduced by talin modulator to inhibit proliferation of human aortic vascular smooth muscle cells^37^.

Besides, these two types of epigenetic regulations also had differences. On one hand, we discovered that either hyper-, hypo- or overall methylation were not related to metabolic pathways, indicating that methylation induced by WEA did not regulate metabolism directly. In addition, it was notable to uncover that WEA induced higher methylation of dsRNA related genes in muscle than that in adipose, indicating functional correlations between dsRNAs and methylation. dsRNAs can be cleaved into small interfering RNAs (siRNA) to trigger RNA interference in plants, animals and some fungi, and is related to methylation. For instance, the formation of dsRNAs for Adar1 binding to Socs3 mRNA is influenced by Tet2 deficiency probably through cytosine methylation-specific readers in mammals^38^. Also, H3 mK9 is the first epigenetic modification made in response to dsRNA-derived species, with DNA methylation accumulating later in the process^39^. Therefore, these dsRNA related DEGs (*TLR3*, *MAPK1*, *DHX15*, *VIM* and *TFRC*) induced by WEA might be essential targets that could also induce downstream DNA methylation and be further investigated to uncover the molecular mechanism in detail. Other methylation specific targets were also enriched in the regulation of GTPase activity, Ras protein signal transduction, positive regulation of NF-kappaB transcription factor activity, transmembrane receptor protein tyrosine kinase activity, etc.

On the other hand, the targets of lncRNAs were specifically enriched in magnesium ion binding, growth hormone receptor binding, lipopolysaccharide receptor activity, transcription factor TFIID complex, etc. Besides, the function of targets of lncRNAs also suggested tissue specificity. Targets were significantly enriched in protein synthesis related GO terms for muscle, e.g., Golgi membrane, endoplasmic reticulum. One metabolism pathway of oxidative phosphorylation was notably specific for adipose. These annotations were distinct between the regulation of lncRNAs in the two tissues.

Another highlight of this work was combining lncRNAs and methylation regulation together to construct the essential epigenetic regulatory network of WEA and tissue differences. In the epigenetic regulatory network of tissue difference, the expressions of all the target genes were lower in muscle than that in adipose, e.g., *ATP1A2*, *CACNA1S*, *CAMK2D*, *ATP2A1* and *RYR1*. ATP1A2 (Na, K-ATPase α2 isoform) is abundantly expressed in skeletal muscle and is regulated by muscle use to maintain contraction and resist fatigue of muscles^40^. *ATP1A2* is mapped to meat quality quantitative trait loci (rs344748241) in a Duroc pig population, demonstrating that *ATP1A2* is highly associated with muscle electric conductivity^41^. Thus, these genes were identified as essential genes to maintain tissue differences under normal conditions by lower expression in these signaling pathways.

As for the epigenetic regulatory network of each tissue, the targets in co-regulated modules all played an important role in signaling transduction, transcription regulation and tissue development. For example, in the co-regulated module of muscle, *MAPKAP1* was such an essential gene that were co-regulated by both lncRNAs and methylation to affect its downstream biological processes, e.g., mTOR signaling pathway, Ras protein signal transduction and regulation of transcription. *FOXP2* was co-regulated with *MAPKAP1*, and was involved in transcription, skeletal muscle tissue development and smooth muscle tissue development. In addition to the importance of these co-regulated targets, our results indicated that these two key genes were regulated by the lncRNAs and methylation regulation that were induced by WEA. Also, *MAPKAP1* and *FOXP2* might had closely functional correlations to reduce meat quality for sheep. Except common signaling pathways, we also noticed that these co-regulated genes (*PDPK1*, *ATP1A2*, *CACNA1S* and *CAMK2D*) were related with Insulin secretion, resistance and signaling pathways, indicating that WEA would also influence insulin to activate downstream processes. Co-regulation of target genes in these modules suggested that WEA induced highly functional correlation of DEGs.

## Conclusions

This study provides a comprehensive overview of biological responses induced by WEA in sheep muscle and adipose. Results suggested that intake of WEA did not directly regulated lipid metabolism but regulated changes of lncRNAs and methylation to affect energy metabolism, signaling pathways and cell proliferation related processes, which were upstream regulating pathways of lipid metabolism. Therefore, this work highlights the underlying molecular mechanism of biological function of *A. mongolicum*, tissue specificity and similarity, as well as the regulation of mutton odor and animal nutrition.

## Methods

### Preparation of water extracts from *A. mongolicum*

*A. mongolicum* powder (Haohai Biotechnology, Inner Mongolia, China) was de-esterified by petroleum ether for 6 h at room temperature. After drying at 65 °C, it was mixed with distilled water at a ratio of 20 ml/g and oscillated by constant temperature water bath shake at 80 °C for 8 h. After filtration, residue was discarded, and solutions were dried in constant temperature drying oven at 65 °C to concentrate to 1/3 of the original volume. Samples were added by anhydrous ethanol of the same volume, kept at 4 °C overnight, and centrifuged at 4000 rpm for 30 min to collect the precipitation. After freeze-drying, water extracts of *A. mongolicum* (WEA) were obtained.

### Feeding and management of experimental animals

All the management and experiments of animals used in the study were approved by Institutional Animal Care and Use Committee of Inner Mongolia Agricultural University and were in accordance with the ARRIVE Guidelines^42^. Experiments were conducted at Fuchuan Feed Science and Technology Co., Ltd. (Bayannaoer, Inner Mongolia, China). Thirty healthy, female, Duhan hybrid meat sheep (4.5-month-old, 36 ± 3.5 kg body weight) were randomly divided into control (B) group and treatment (N) group equally. Sheep were fed with basal diets and mixture of basal diets and WEA (3.4 g per day per animal). Experiments lasted for 75 days (from September 10 to November 24, 2017) including a 15-day preliminary feeding period for adaptation and a 60-day experimental feeding period. Experimental sheep were fed twice a day. Water and food were available ad libitum*.* Compositions of basal diet were listed in Table S7, and nutrient levels of basal diet were listed in Table S8.

After 24 h pre-slaughter fasting time, 6 animals were randomly selected from B and N groups and sent to the slaughterhouse. Longissimus dorsi muscle (M) and subcutaneous fat (F) were immediately taken after slaughter and washed with normal saline. Tissue samples (BM, BF, NM and NF) were cut into small pieces and packed into multiple cryopreservation tubes respectively and stored in liquid nitrogen and transported by dry ice.

### RNA isolation, library preparation and sequencing

Total RNA was extracted from sheep muscle and adipose using Trizol reagent (Life Technologies, CA, USA). We examined RNA purity, concentration and integrity using NanoPhotometer spectrophotometer (IMPLEN, CA, USA), Qubit RNA Assay Kit (Life Technologies, CA, USA) and RNA Nano 6000 Assay Kit (Agilent Technologies, CA, USA). Ribosomal RNA and rRNA free residue were removed from 3 μg RNA per sample by Epicentre Ribo-zero rRNA Removal Kit (Epicentre, USA) and ethanol precipitation. Sequencing libraries were generated using rRNA depleted RNA by NEBNext Ultra Directional RNA Library Prep Kit for Illumina (NEB, USA) and sequenced on an Illumina Hiseq 4000platform and 150 bp paired-end reads were generated.

### Data analyses of RNA sequencing

Reads containing adapter and containing ploy-N and low-quality reads were removed from raw reads to obtain clean reads by PERL. Q20, Q30 and GC content of clean data were calculated. All downstream analyses were based on clean data with high quality. Reference genome and gene model annotation files were referred to Ovis aries Oar_rambouillet_v1.0. Reference genome was indexed using bowtie2 v2.2.8, while paired-end clean reads were aligned to reference genome using HISAT2 (v2.0.4) with default parameters. Mapped reads were assembled by StringTie (v1.3.1)^43^ in a reference-based approach. Principle component analyses (PCA) was performed in FactoMineR (v2.4) in R^44^.

### Identification of lncRNAs

Transcripts with exon number ≥ 2 and length > 200 bp were maintained. Transcripts that have overlap regions with annotated exon region were filtered by Cuffcompare in Cufflinks software (v2.2.0)^45^. Expressions of transcripts were computed by Cuffquant in Cufflinks^45^ and transcripts with FPKM ≥ 0.5 were kept for further analyses. Coding potential of transcripts were measured by CNCI (v2)^46^, CPC2 (v0.9-r2)^47^, Pfam Scan (v1.3)^48,49^ and PhyloCSF (v20121028)^50^. Transcripts predicted with coding potential by the four software were filtered out, and the rest without coding potential were candidate lncRNAs.

### Quantification of expressions and computation of differential expression

Cuffdiff in Cufflinks^45^ was used to calculate FPKMs of lncRNAs and coding genes. Gene FPKMs were computed by summing the FPKMs of transcripts in each gene group. Ballgown suite includes functions for interactive exploration of the transcriptome assembly, visualization of transcript structures and feature-specific abundances for each locus, and post-hoc annotation of assembled features to annotated features^51^. Transcripts with P-adjust < 0.05 were defined as differentially expressed. Expression profiles of DEGs were illustrated by Heatmap using pheatmap R package^52^.

### Prediction of target genes of lncRNAs

Potential target genes of lncRNAs were predicted by co-location and co-expression. For co-location, target genes were defined as the genes located within the region between 100 kb up- and down-stream of lncRNAs. For co-expression, lncRNAs were co-expressed with their target genes. Expression correlation of lncRNAs and genes was computed and Pearson correlation co-efficient (PCC) was applied. P-values were adjusted by Benjamini and Hochberg false discovery rate. Co-expressed lncRNAs and genes were defined as the pairs with |PCC|> 0.95 and adjust *P* value < 0.05. Potential target genes were defined as genes satisfied with either one of two criterions.

### Library preparation and quantification for Bisulfite sequencing

Total DNA was extracted from muscle and adipose using Trizol (TaKaRa Biotechnology Co., Ltd.). Genomic DNA purity and concentration was measured using the NanoPhotometer spectrophotometer (IMPLEN, CA, USA) and Qubit DNA Assay Kit in Qubit 2.0 Flurometer (Life Technologies, CA, USA) respectively. Bisulfite sequencing was performed as published procedures^53^, 5.2 µg genomic DNA spiked with 26 ng lambda DNA were fragmented by sonication to 200–300 bp with Covaris S220, followed by end repair and adenylation. Cytosine-methylated barcodes were ligated to sonicated DNA. DNA fragments were treated twice with bisulfite using EZ DNA Methylation-GoldTM Kit (Zymo Research), before resulting single-strand DNA fragments were PCR amplificated using KAPA HiFi HotStart Uracil and ReadyMix (2X). Library concentration was quantified, and insert size was assayed on Agilent Bioanalyzer 2100 system. Library preparations were sequenced on an Illumina Hiseq 4000 platform and 150 bp paired-end reads were generated.

### Processing of methylation sequencing data

FastQC (v0.11.5)^54^ was used to perform basic statistics on read quality. Reads sequences in FASTQ format were pre-processed through Trimmomatic (v0.36)^55^. Remaining reads were counted as clean reads for all subsequent analyses. Bismark software (v0.16.3)^56^ was used to perform alignments to a reference genome. Reference genome was transformed into bisulfite-converted version and indexed using bowtie2^57^. Reads were transformed into fully bisulfite-converted versions. Sequence reads that produced a unique best alignment from the two alignment processes were compared to genomic sequence and methylation state of all cytosine positions in the reads was inferred. Sequencing depth and coverage were summarized using deduplicated reads. Sodium bisulfite non-coversion rate was calculated as the percentage of cytosine sequenced at cytosine reference positions.

### Estimation of methylation levels and differentially methylated regions (DMRs)

To identify methylation site, we modeled sum Mc of methylated counts as a binomial (Bin) random variable with methylation rate r:

$$ {\mathbf{m}}{\varvec{C}}\sim{\varvec{Bln}}({\varvec{mC}} + \user2{umC*r} $$

We divided sequence into 10 kb bins. and calculated sum of methylated and unmethylated reads counts in each window. Methylation level (ML) for each window or C site was fraction of methylated Cs:

$$ {\varvec{ML}}\left( {\varvec{C}} \right) = \frac{{{\varvec{reads}}\left( {{\varvec{mC}}} \right)}}{{{\varvec{reads}}\left( {{\varvec{mC}}} \right) + {\varvec{reads}}\left( {\varvec{C}} \right)}} $$

Calculated ML was corrected with the bisulfite non-conversion rate according to previous studies^58^. Given bisulfite non-conversion rate r, corrected ML was estimated as:

$$ {\varvec{ML}}_{{\left( {{\varvec{corrected}}} \right)}} = \frac{{{\varvec{ML}} - {\varvec{r}}}}{{1 - {\varvec{r}}}} $$

Differentially methylated regions (DMRs) were identified using the DSS software^59–61^. According to distribution of DMRs through genome, we defined DMRs genes whose gene body region (from TSS to TES) or promoter region (upstream 2 kb from the TSS) had an overlap with the DMRs.

### Pathway and GO enrichment analyses

Enrichment analyses measure the relationship between KEGG^62^ and GO annotation from Uniprot Database^63^ for desired gene sets. Desired gene sets were target genes denoted by G(g). Enrichment analysis was tested by hypergeometric distribution, and *P* value were correlated by Benjamini and Hochberg false discovery rate controlling procedure. Enriched KEGG pathways or GO terms were defined as the ones with adjust *P* value less than 0.01. Statistics were analyzed by R.

### Construction of regulatory network for epigenetics

Target genes of both DMRs and lncRNAs were maintained to construct regulatory networks of epigenetics for tissue difference and individual tissue that were affected by WEA. Transcription expression of target genes and lncRNAs were both identified as differentially expressed for BM-BF, NM-NF, NM-BM and NF-BF. Regulatory networks of epigenetics were presented by Cytoscape software^64^. Node degree was computed by Cytoscape. Co-regulated modules were defined as targets that were regulated by the same lncRNAs.

### Validation of essential target genes by qRT-PCR

RNA quality was assessed by electrophoresis (1% agarose gels) before reverse transcription into cDNA (TaKaRa Biotechnology Co., Ltd.). Gene primers (Table S9) were designed by Primer 6.0 (PE Applied Biosystems, Inc., Foster City, CA). mRNA expression was estimated by qRT-PCR analysis using FS Universal SYBR Green Real Master (Roche, Indianapolis, IN) on 7500 Real-Time PCR System (Applied Biosystems). Conditions were as follows: 95 °C for 3 min, followed by 40 cycles of 95 °C for 15 s and 60 °C for 30 s. All reactions were run in triplicate. Relative expression levels were normalized by β-actin.

### Ethics approval and consent to participate

In this study, experimental environment followed standards relevant to an ordinary animal laboratory facility in China National Standard “Laboratory animal environment and facilities” (GB14925-2010). The feeding and the experimental operations on animals were in accordance with the animal welfare requirements.

## Supplementary Information

Supplementary Figure S1.

Supplementary Figure S2.

Supplementary Figure S3.

Supplementary Figure S4.

Supplementary Figure S5.

Supplementary Figure S6.

Supplementary Figure S7.

Supplementary Figure S8.

Supplementary Figure S9.

Supplementary Figure S10.

Supplementary Figure S11.

Supplementary Figure S12.

Supplementary Figure S13.

Supplementary Figure S14.

Supplementary Figure S15.

Supplementary Figure S16.

Supplementary Figure S17.

Supplementary Tables.

## Data Availability

Raw data can be accessed by NCBI BioProject database with identifier PRJNA656426.
