# Supplementary material for: Tissue-specific regulatory mechanism of LncRNAs and methylation in sheep adipose and muscle induced by Allium mongolicum Regel extracts
Source: Sci Rep. 2021 Apr 28;11:9186. doi: 10.1038/s41598-021-88444-9 (PMC8080592; doi:10.1038/s41598-021-88444-9)
Supplement: Supplementary file 1 — Supplementary Figure S1. [file 41598_2021_88444_MOESM1_ESM.pdf]

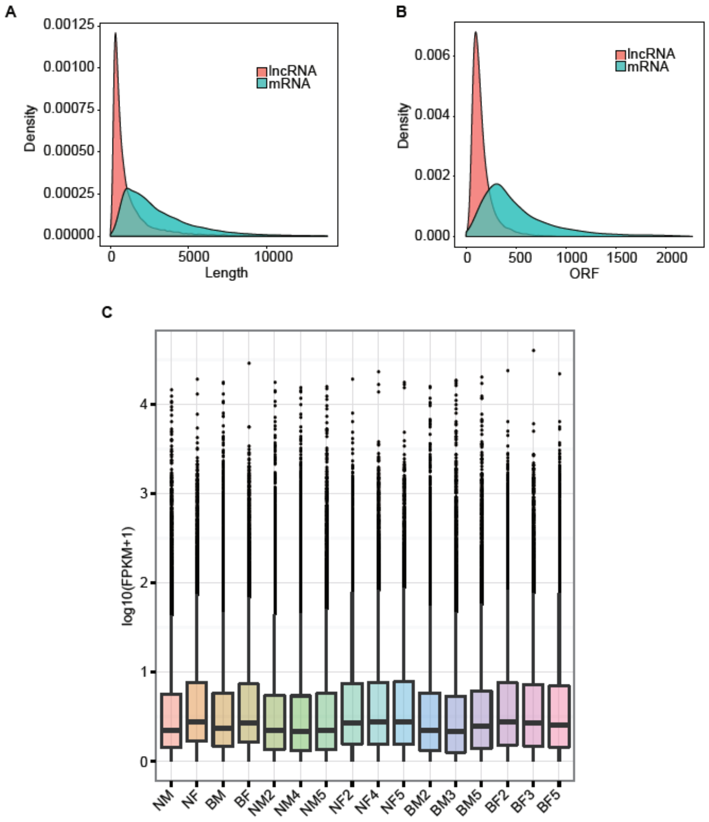

Figure S1. Expression analyses for lncRNA and mRNA. **a**, length densities of lncRNA and mRNA. **b**, ORF density of lncRNA and mRNA. **c**, box plot of FPKM distribution of RNA-seq results in all samples.
