# Supplementary figures and images for: Tissue-specific regulatory mechanism of LncRNAs and methylation in sheep adipose and muscle induced by Allium mongolicum Regel extracts
Source: Sci Rep. 2021 Apr 28;11:9186. doi: 10.1038/s41598-021-88444-9 (PMC8080592; doi:10.1038/s41598-021-88444-9)

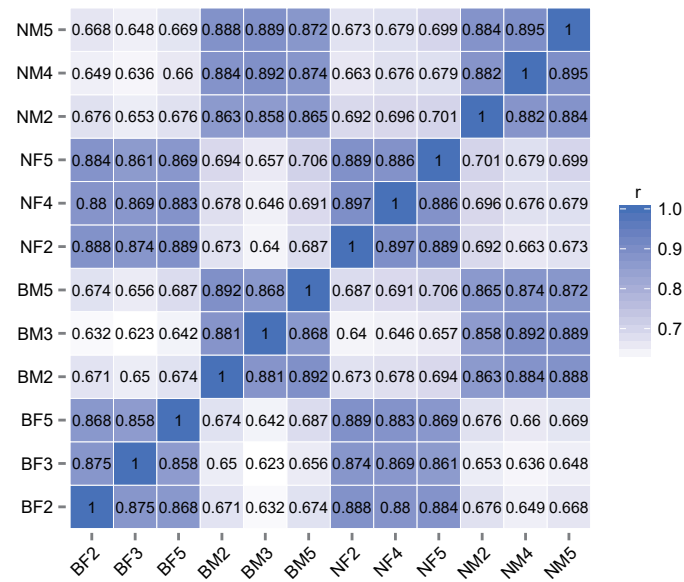

**Figure S2.** Person correlation coefficients among samples computed by genome FPKM.

Supplement: Supplementary file 2 — Supplementary Figure S2. [file 41598_2021_88444_MOESM2_ESM.pdf]

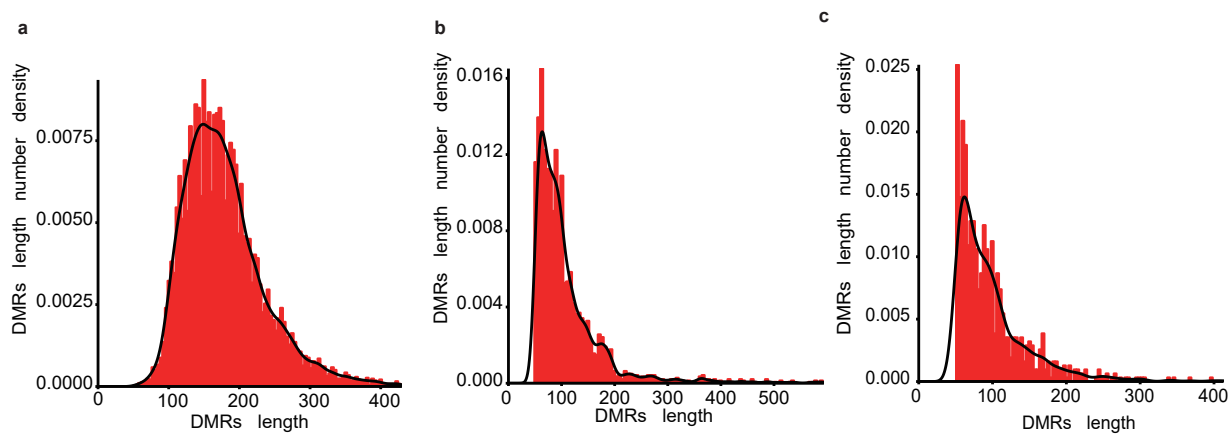

**Figure S5.** Density distribution of CG (a), CHG (b) and CHH (c) DMRs length numbers in adipose.

Supplement: Supplementary file 5 — Supplementary Figure S5. [file 41598_2021_88444_MOESM5_ESM.pdf]

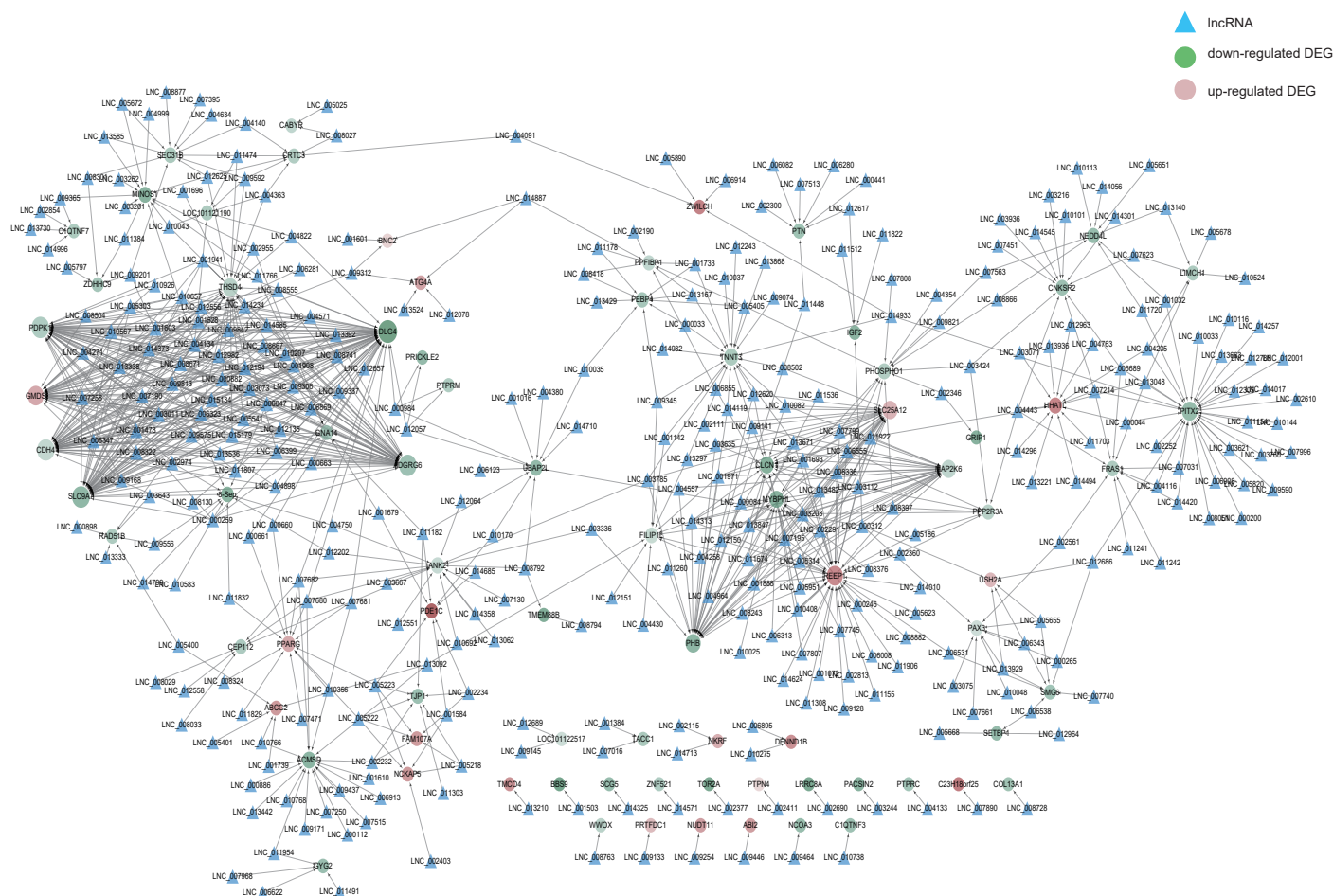

**Figure S16.** The co-regulatory network of both lncRNA and methylation for muscle induced by WEA.

Supplement: Supplementary file 16 — Supplementary Figure S16. [file 41598_2021_88444_MOESM16_ESM.pdf]

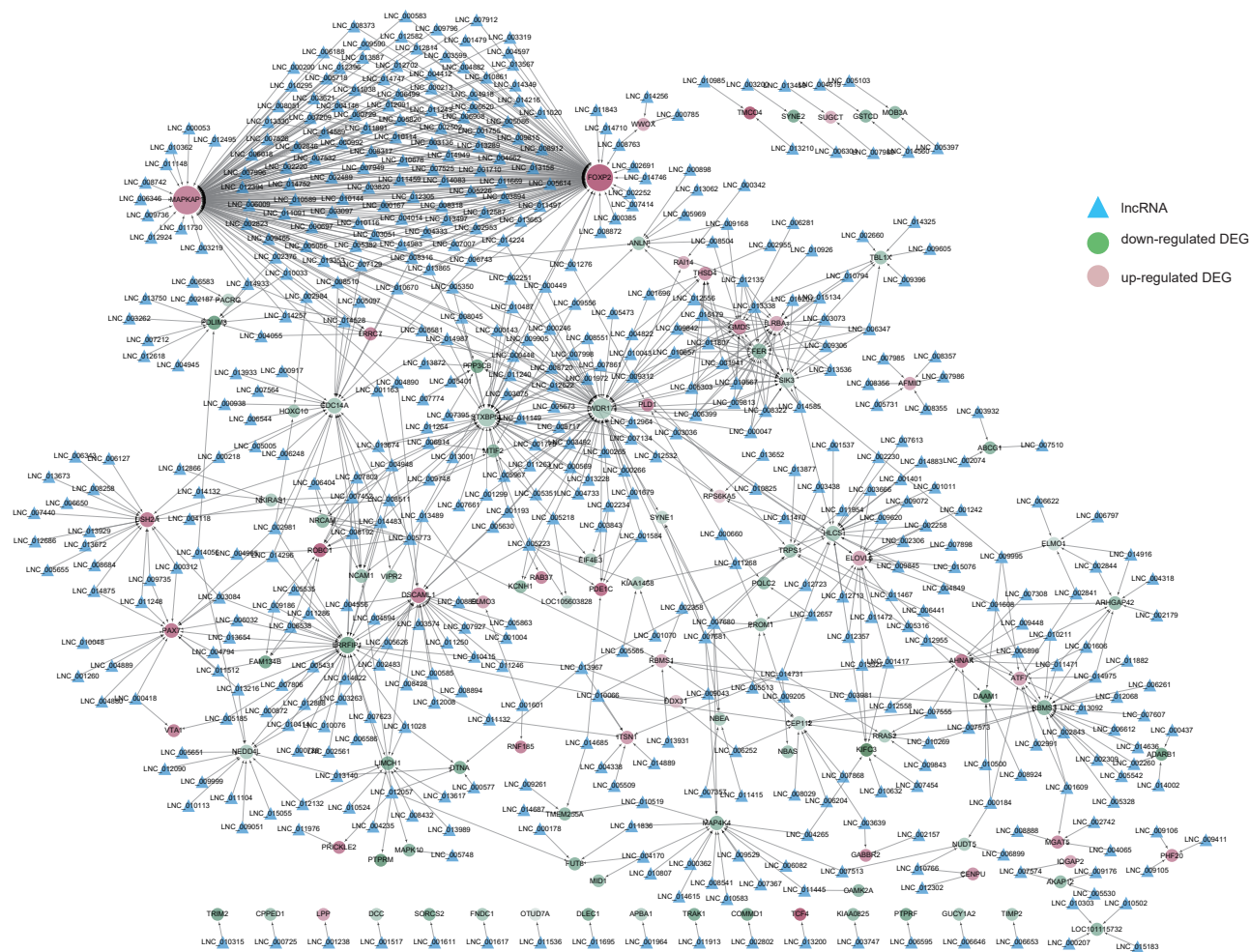

**Figure S17.** The co-regulatory network of both lncRNA and methylation for adipose induced by WEA.

Supplement: Supplementary file 17 — Supplementary Figure S17. [file 41598_2021_88444_MOESM17_ESM.pdf]
