# Supplementary material for: Tissue-specific regulatory mechanism of LncRNAs and methylation in sheep adipose and muscle induced by Allium mongolicum Regel extracts
Source: Sci Rep. 2021 Apr 28;11:9186. doi: 10.1038/s41598-021-88444-9 (PMC8080592; doi:10.1038/s41598-021-88444-9)
Supplement: Supplementary file 6 — Supplementary Figure S6. [file 41598_2021_88444_MOESM6_ESM.pdf]

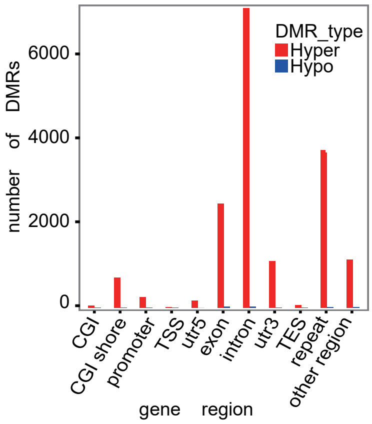

Figure S6. Numbers of hyper and hypo CHH DMRs on CGI, CGI shore, TSS, utr5, exon, intron, utr3, TES, repeat and other regions for adipose.
