# Supplementary material for: Tissue-specific regulatory mechanism of LncRNAs and methylation in sheep adipose and muscle induced by Allium mongolicum Regel extracts
Source: Sci Rep. 2021 Apr 28;11:9186. doi: 10.1038/s41598-021-88444-9 (PMC8080592; doi:10.1038/s41598-021-88444-9)
Supplement: Supplementary file 7 — Supplementary Figure S7. [file 41598_2021_88444_MOESM7_ESM.pdf]

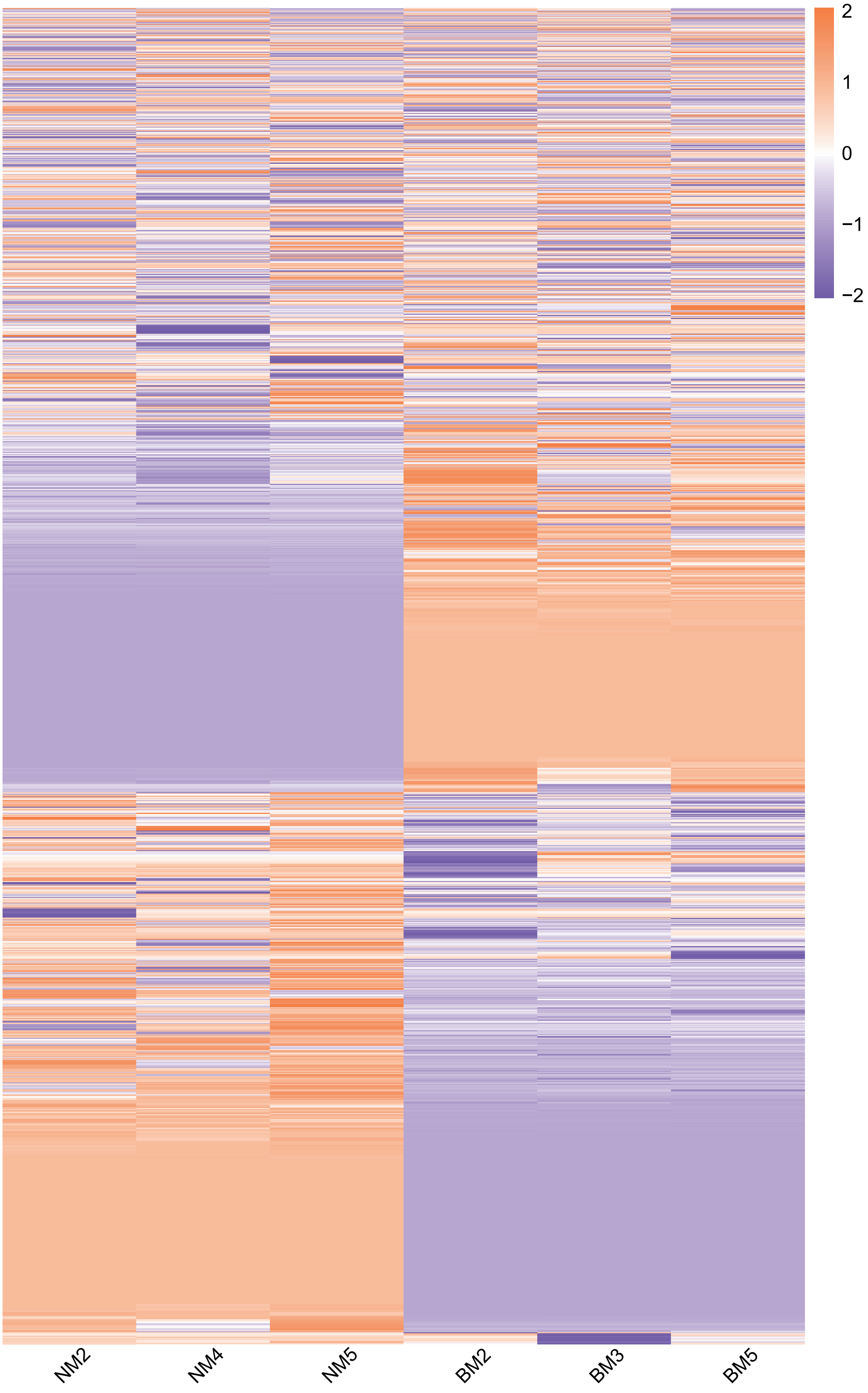


**Figure S7**. Expression profiles of differentially expressed genes in normal muscle (BM) and muscle tissues treated with WEA (NM).
