# Supplementary material for: Tissue-specific regulatory mechanism of LncRNAs and methylation in sheep adipose and muscle induced by Allium mongolicum Regel extracts
Source: Sci Rep. 2021 Apr 28;11:9186. doi: 10.1038/s41598-021-88444-9 (PMC8080592; doi:10.1038/s41598-021-88444-9)
Supplement: Supplementary file 8 — Supplementary Figure S8. [file 41598_2021_88444_MOESM8_ESM.pdf]

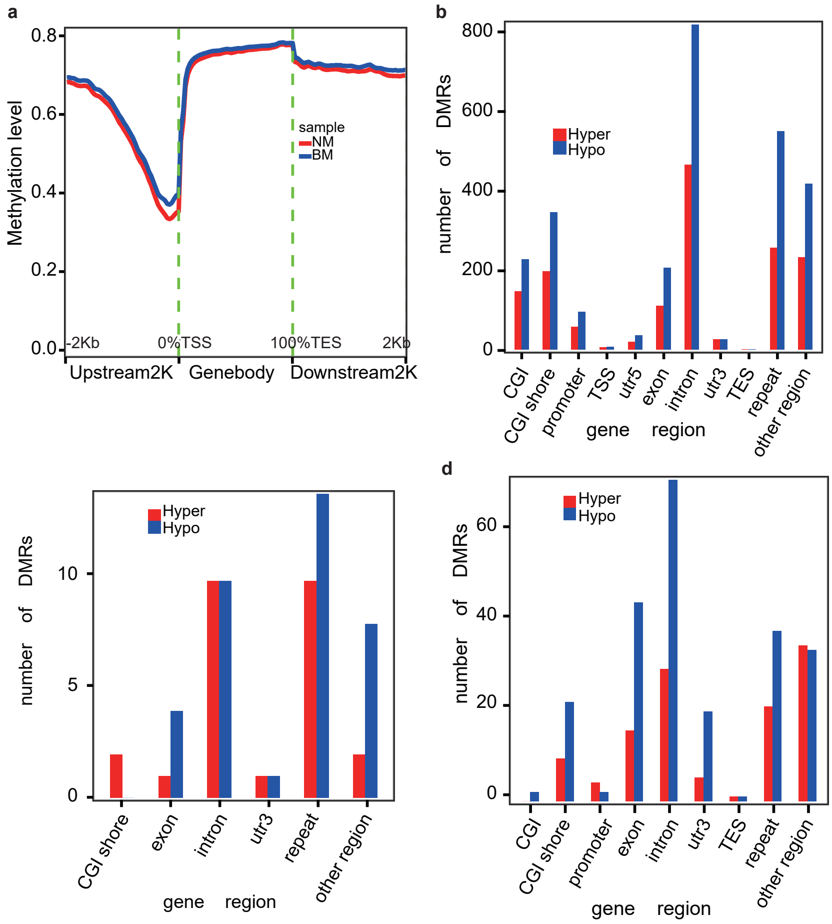

Figure S8. Effects of WEA on methylation levels and DMRs in muscle. **a**, whole genome methylation levels from upstream 2K of TSS to downstream 2K of TES in muscle. CG (**b**), CHG (**c**) and CHH (**d**) methylation levels against CGI, CGI shore, promoter, utr5, exon, intron, utr3 and repeat regions in muscle.
