# Supplementary material for: Tissue-specific regulatory mechanism of LncRNAs and methylation in sheep adipose and muscle induced by Allium mongolicum Regel extracts
Source: Sci Rep. 2021 Apr 28;11:9186. doi: 10.1038/s41598-021-88444-9 (PMC8080592; doi:10.1038/s41598-021-88444-9)
Supplement: Supplementary file 11 — Supplementary Figure S11. [file 41598_2021_88444_MOESM11_ESM.pdf]

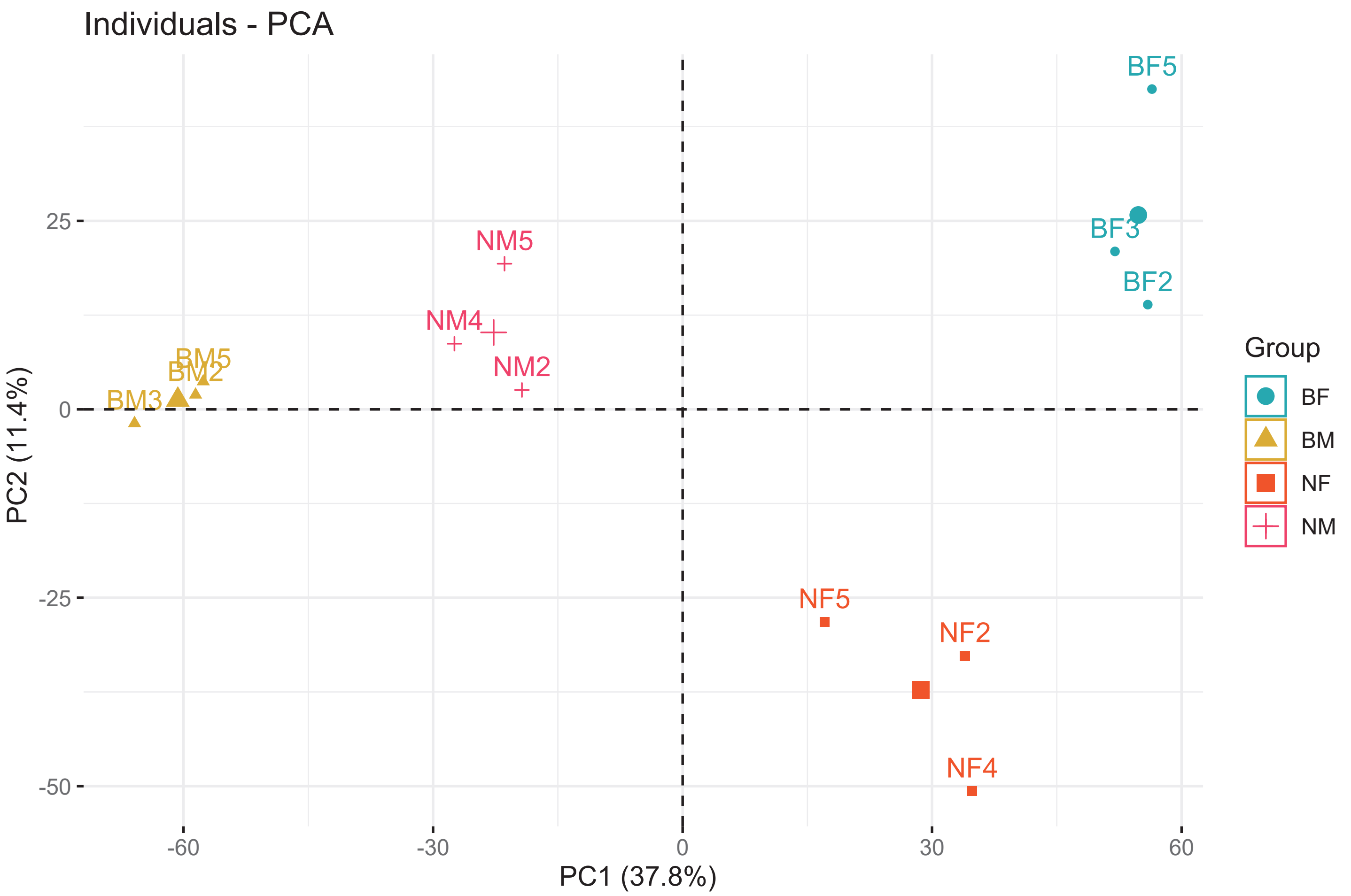

Figure S11. Principal component analysis (PCA) score plot for transcriptome of 12 samples from normal adipose (BF), normal muscle(BM), and treated adipose (NF) and muscle tissues (NM).
