# Supplementary material for: Tissue-specific regulatory mechanism of LncRNAs and methylation in sheep adipose and muscle induced by Allium mongolicum Regel extracts
Source: Sci Rep. 2021 Apr 28;11:9186. doi: 10.1038/s41598-021-88444-9 (PMC8080592; doi:10.1038/s41598-021-88444-9)
Supplement: Supplementary file 12 — Supplementary Figure S12. [file 41598_2021_88444_MOESM12_ESM.pdf]

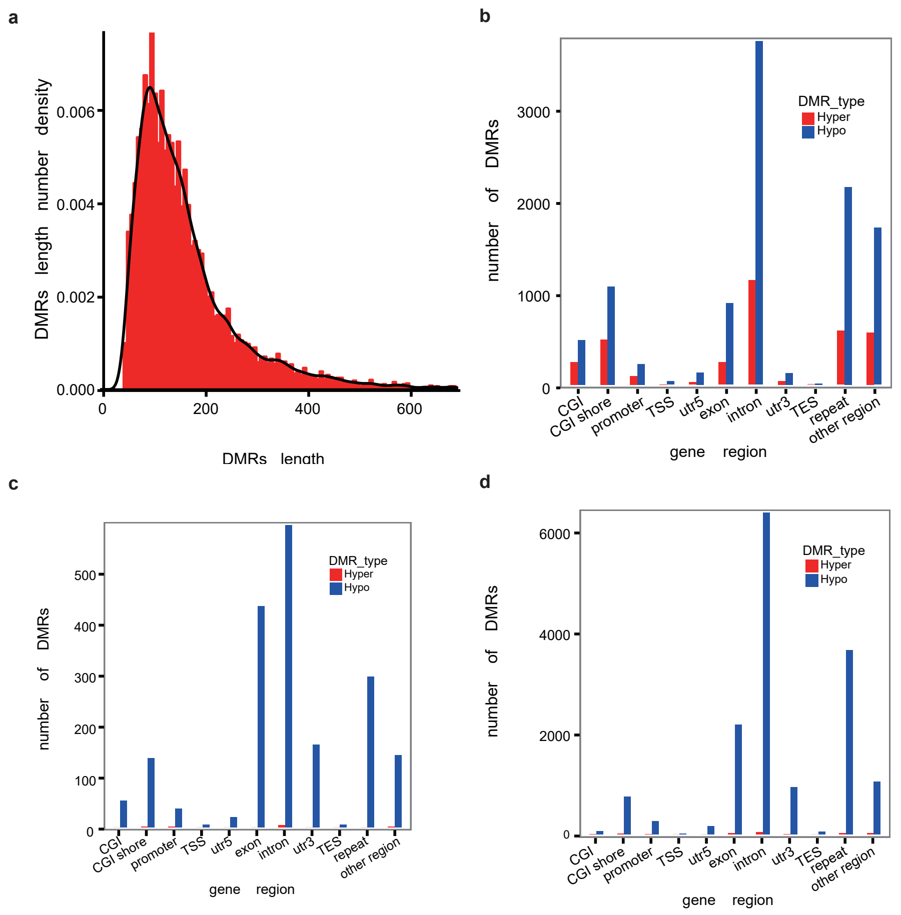

Figure S12. Effects of WEA on whole genome methylation DMRs length and numbers between adipose and muscle. **a**, density distribution of DMRs length numbers. DMR numbers of CG (**b**), CHG (**c**) and CHH (**d**) against CGI, CGI shore, promoter, utr5, exon, intron, utr3 and repeat regions in tissue difference induced by WEA.
