# Supplementary material for: Tissue-specific regulatory mechanism of LncRNAs and methylation in sheep adipose and muscle induced by Allium mongolicum Regel extracts
Source: Sci Rep. 2021 Apr 28;11:9186. doi: 10.1038/s41598-021-88444-9 (PMC8080592; doi:10.1038/s41598-021-88444-9)
Supplement: Supplementary file 13 — Supplementary Figure S13. [file 41598_2021_88444_MOESM13_ESM.pdf]

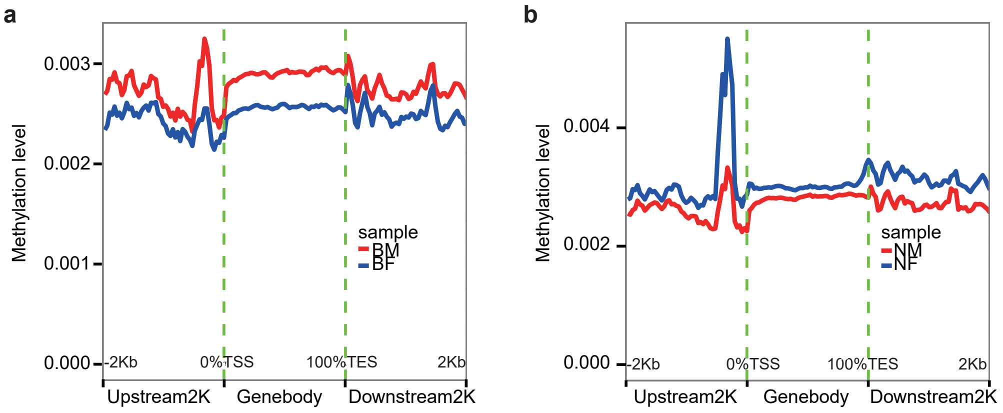

Figure S13. Comparisons of whole genome methylation levels from upstream 2K of TSS to downstream 2K of TES for normal muscle and adipose (**a**), and tissues induced by WEA (**b**).
