# Supplementary material for: Tissue-specific regulatory mechanism of LncRNAs and methylation in sheep adipose and muscle induced by Allium mongolicum Regel extracts
Source: Sci Rep. 2021 Apr 28;11:9186. doi: 10.1038/s41598-021-88444-9 (PMC8080592; doi:10.1038/s41598-021-88444-9)
Supplement: Supplementary file 14 — Supplementary Figure S14. [file 41598_2021_88444_MOESM14_ESM.pdf]

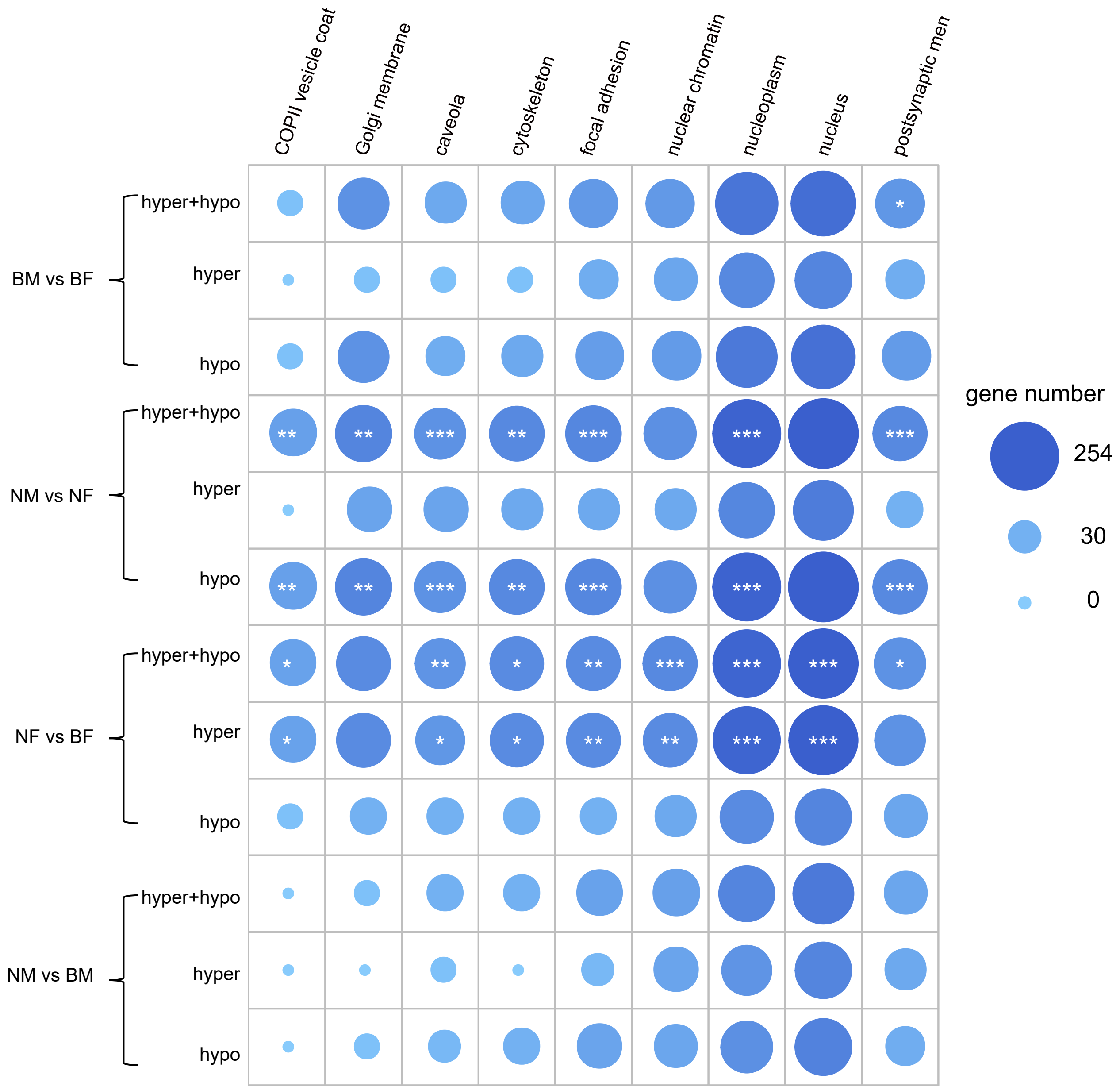

Figure S14. 9 enriched cellular component GO terms of different methylation levels for normal tissue comparison (BM vs BF), tissue comparison induced by WEA (NM vs NF), effects of WEA on adipose (NF vs BF) and muscle (NM vs BM). * p≤ 0.05, ** p≤ 0.01, *** p≤ 0.001
