# Supplementary material for: Tissue-specific regulatory mechanism of LncRNAs and methylation in sheep adipose and muscle induced by Allium mongolicum Regel extracts
Source: Sci Rep. 2021 Apr 28;11:9186. doi: 10.1038/s41598-021-88444-9 (PMC8080592; doi:10.1038/s41598-021-88444-9)
Supplement: Supplementary file 15 — Supplementary Figure S15. [file 41598_2021_88444_MOESM15_ESM.pdf]

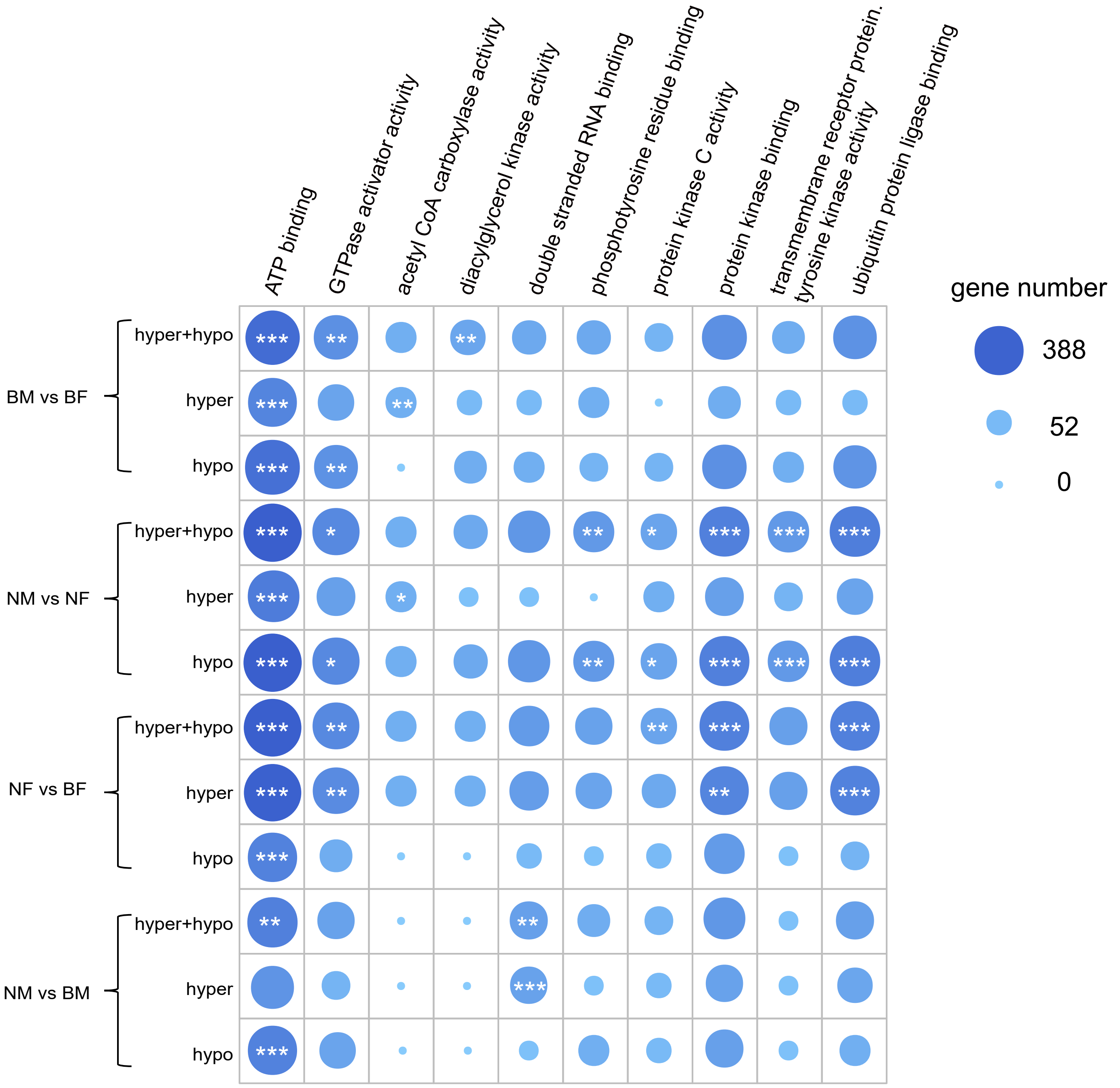


**Figure S15**. 10 enriched molecular function GO terms of different methylation levels for normal tissue comparison (BM vs BF), tissue comparison induced by WEA (NM vs NF), effects of WEA on adipose (NF vs BF) and muscle (NM vs BM). * p≤ 0.05, ** p≤ 0.01, *** p≤ 0.001
